# Pacidusin B isolated from *Phyllanthus acidus* triggers ferroptotic cell death in HT1080 cells

**DOI:** 10.1007/s13659-024-00454-y

**Published:** 2024-05-23

**Authors:** Guangyu Zhu, Dian Luo, Yueqin Zhao, Zhengrui Xiang, Chao Chen, Na Li, Xiaojiang Hao, Xiao Ding, Yingjun Zhang, Yuhan Zhao

**Affiliations:** 1 State Key Laboratory of Phytochemistry and Plant Resources in West China, Kunming Institute of Botany, Chinese Academy of Sciences, Kunming, 650201 China; 2 University of Chinese Academy of Sciences, Beijing, 100049 China; 3 Department of Orthopedics, The First Affiliated Hospital of Kunming Medical University, Kunming, 650032 China; 4 Research Unit of Chemical Biology of Natural Anti-Virus Products, Chinese Academy of Medical Sciences, Beijing, 100730 China; 5 Yunnan Key Laboratory of Natural Medicinal Chemistry, Kunming Institute of Botany, Chinese Academy of Sciences, Kunming, 650201 China

**Keywords:** Ferroptosis, Pacidusin B, ER stress, PERK-Nrf2-HO-1 pathway, xCT

## Abstract

Cancer cells generally exhibit ‘iron addiction’ phenotypes, which contribute to their vulnerability to ferroptosis inducers. Ferroptosis is a newly discovered form of programmed cell death caused by iron-dependent lipid peroxidation. In the present study, pacidusin B, a dichapetalin-type triterpenoid from *Phyllanthus acidus* (L.) Skeels (Euphorbiaceae), induces ferroptosis in the HT1080 human fibrosarcoma cell line. Cells treated with pacidusin B exhibited the morphological characteristic ‘ballooning’ phenotype of ferroptosis. The biochemical hallmarks of ferroptosis were also observed in pacidusin B-treated cells. Both oxidative stress and ER stress play significant roles in pacidusin B-induced ferroptosis. The activation of the PERK-Nrf2-HO-1 signaling pathway led to iron overload, while inhibition of GPX4 further sensitized cancer cells to ferroptosis. Furthermore, the molecular docking study showed that pacidusin B docked in the same pocket in xCT as the ferroptosis inducer erastin. These results revealed that pacidusin B exerts anticancer effects via inducing ER-mediated ferroptotic cell death.

**Graphical Abstract:**

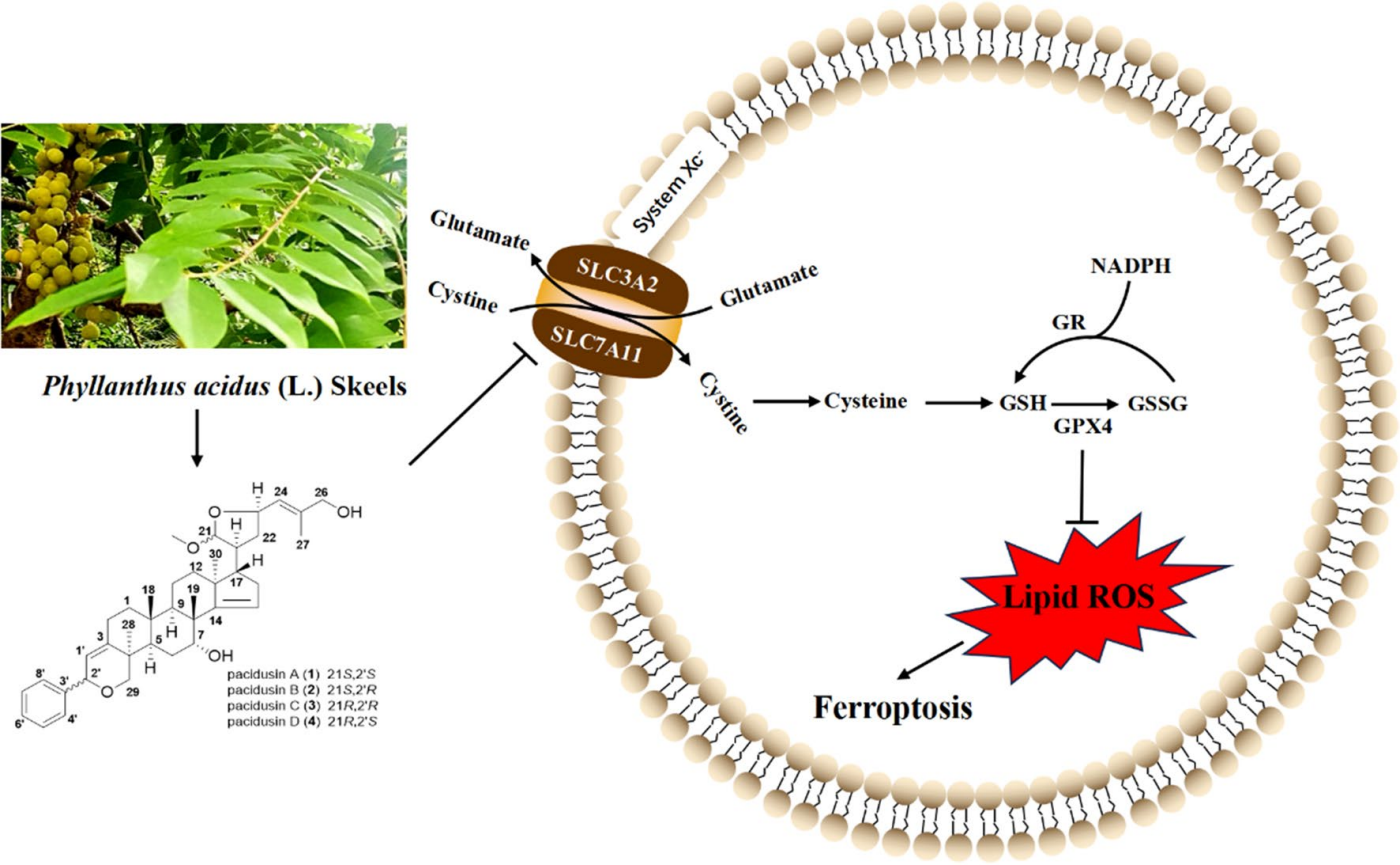

## Introduction

In recent decades, many modes of programmed cell death other than apoptosis have been characterized. In 2003, erastin was reported to exhibit a selective lethal effect on cancer cells expressing RAS, and the mode of cell death was different from that described previously [1]. Until 2012, this kind of cell death, mediated mainly by the iron-catalyzed Fenton reaction of polyunsaturated fatty acids (PUFAs), was coined as ferroptosis [2]. One of the morphological characteristics observed during ferroptosis was the obvious ‘ballooning’ phenotype, which may be caused by the disruption of membrane stabilization and cytoskeleton rearrangement [3]. There are three hallmarks of ferroptosis: the accumulation of redox-active iron, the oxidation of PUFA-containing phospholipids, and the loss of lipid peroxide repair capacity by glutathione peroxidase 4 (GPX4). GPX4 is a selenoprotein that protects cells from damage by catalyzing the reduction of H_2_O_2_ and hydroperoxides [4, 5]. The system Xc^−^/glutathione (GSH)/GPX4 axis is one of the most important antioxidant systems in ferroptotic cells.System Xc^−^, a cystine/glutamate transporter, is a dimer composed of solute carrier family 3 member 2 (SLC3A2) and solute carrier family 7 member 11 (SLC7A11, also known as xCT). Cystine imported from the extracellular space by system Xc^−^ is reduced to cysteine, which serves as a precursor for the synthesis of GSH [6]. GSH is a tripeptide antioxidant that is widely distributed in cells and controls intracellular oxidation levels by acting as an iron chelator or a cofactor for GPX4 [7–10]. Two well-known ferroptosis inhibitors, erastin and RSL3, function mainly through the inhibition of xCT or GPX4 [3, 11]. Recent studies have shown that xCT is highly expressed in a variety of human cancer types and plays a crucial role in the survival, growth and malignant progression of tumor cells, which makes it a promising therapeutic target for cancer [12–15].

Iron overload or elevated levels of Labile iron pools (LIPs) also contribute to ferroptosis. Iron has redox activity and can participate in the formation of free radicals and lipid peroxidation. LIPs are a collection of redox-active iron complexes, most of which are Fe^2+^. Fe^2+^ is oxidized in the Fenton reaction and triggers lipid peroxidation [7, 16]. It has been reported that cancer cells display impaired iron homeostasis. Additional iron is needed for the growth and metabolism of cancer cells [17]. Iron levels are increased in cancer cells by dysregulation of iron uptake genes or iron-regulating genes, which contributes to the so-called ‘iron addiction’ phenotype of malignant cancer cells [18]. Targeting iron metabolic pathways or iron-dependent ferroptosis may provide a new strategy for cancer therapy [7]. Many natural small molecule compounds, such as FINO_2_ [19], withaferin A [20], and tagitinine C [21] have been reported to induce ferroptotic cell death by targeting iron metabolism.

As ferroptosis is a unique form of cell death that is mechanistically and morphologically different from other forms of cell death, targeting ferroptosis has been considered a potential therapeutic strategy in cancer treatment [22–24]. In addition, extensive studies have shown that inducing ferroptosis in tumor cells can reverse drug resistance [25]. Thus, ferroptosis inducers (FINs) hold great potential in cancer therapy. FINs are divided into four categories according to the targets of inducers, including inducers targeting the GSH/GPX4 axis, lipid metabolism, iron metabolism and ferroptosis-suppressor-protein 1 (FSP1)/CoQ-related pathways [26]. Many naturally derived small molecules have been reported to induce ferroptosis, providing novel mechanisms regulating ferroptosis and potential lead compounds targeting ferroptosis for cancer therapy.

*Phyllanthus acidus* (L.) Skeels is a tropical plant distributed widely in many Southeast Asian and South Asian countries, such as Thailand, Malaysia, Indonesia, Vietnam, Laos, and India, and has been introduced and cultivated in the tropical areas of Yunnan Province, China [27, 28]. The bark, roots and leaves of *P. acidus*, which contain mainly flavonoids, terpenoids, organic acids, nucleosides, amino acid derivatives, and phenolic compounds [29–36], have been used as folk medicine to treat cough, rheumatic low back pain, bronchitis, and other symptoms by the local people of its growing areas. Tender leaves are also utilized as edible vegetables in Indonesia, Thailand, and India, and infusions are normally used as dieting aids for people trying to lose weight [28]. Compounds isolated from *P. acidus* showed antibacterial, anti-inflammatory, hepatoprotective, hypoglycemic and hypotensive activities [27, 30, 37]. Among these compounds, diterpenoids and dichapetalin triterpenoids isolated from *P. acidus* displayed cytotoxicity to tumor cells, suggesting their potential as anticancer drugs [29, 30]. In view of the antitumor activity of *P. acidus*, we further evaluated whether dichapetalin triterpenoids induced ferroptosis.

## Results

### Pacidusin B inhibits cell proliferation and induces cell death

**Fig. 1:**
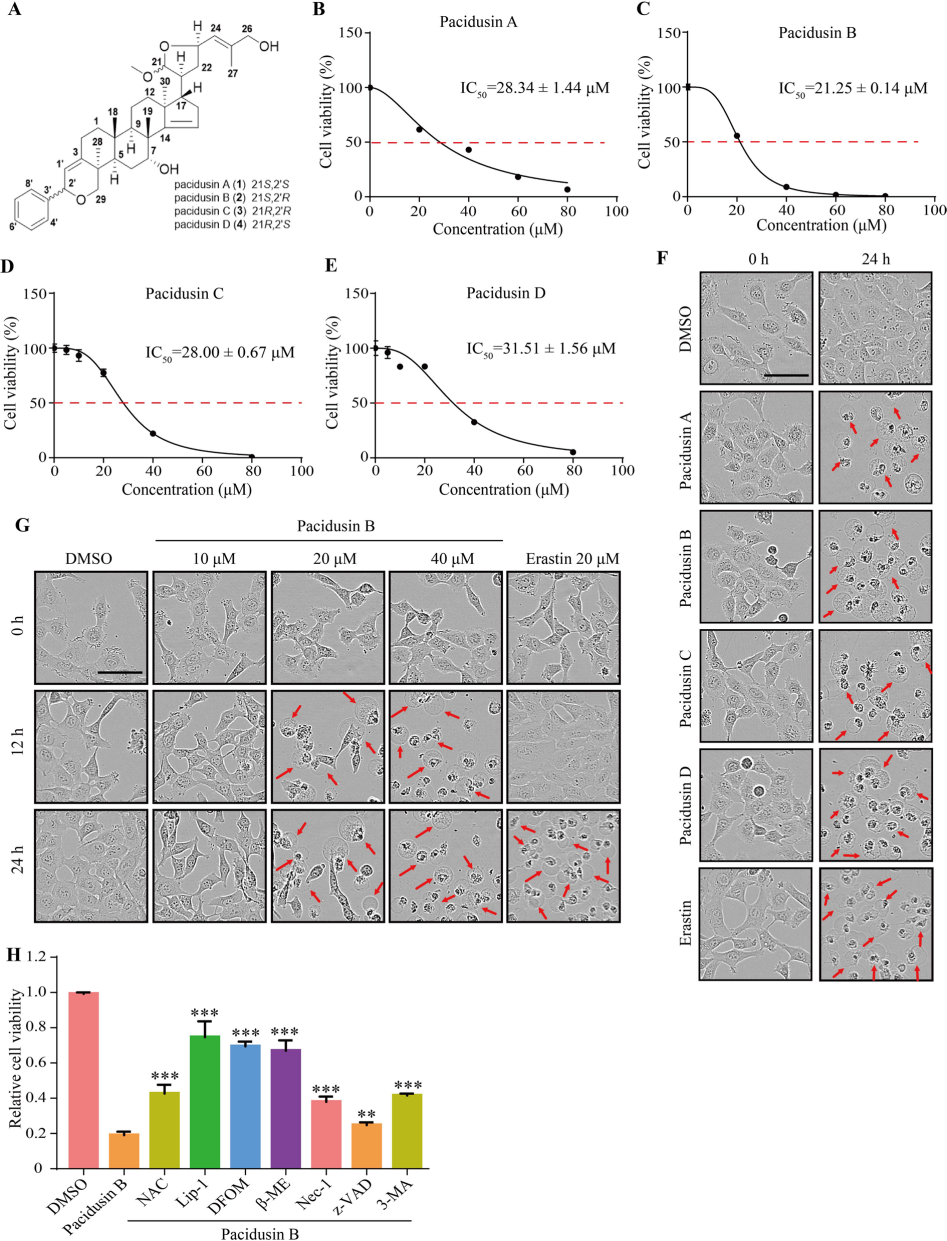
Pacidusin B inhibits cell proliferation and induces cell death. **A** The chemical structures of pacidusins A-D. **B–E** An MTT assay was used to determine the viability of HT1080 cells treated with the specified concentrations of the test compounds for 24 h. **F** Morphology of HT1080 cells treated with pacidusins A-D (40 μM) for 24 h. Scale bar, 50 μm. **G** Morphology of HT1080 cells treated with pacidusin B (10, 20, and 40 μM) for 24 h. Scale bar, 50 μm. **H** Effect of treatment with pacidusin B in combination with other cell death inhibitors on HT1080 cells. The data are presented as the mean ± SD. **p < 0.01, ***p < 0.001

Four dichapetalin triterpenoids (pacidusins A–D, purity > 95%) were extracted and purified from the leaves of *P. acidus* (Euphorbiaceae) [30], and the structures of these compounds are shown in Fig. 1A. The cytotoxic effects of these four compounds on the proliferation of HT1080 cells were evaluated and the IC_50_ values of each compound were determined (Fig. 1B–E). Pacidusin B, which was selected for subsequent studies, showed the most potent cytotoxic effect on HT1080 cells.

In addition, the pacidusin A-D-treated cells exhibited a ‘ballooning’ phenotype [3], which is a morphological feature of cells during ferroptosis (Fig. 1F). The morphologicial changes of pacidusin B-treated cells were further evaluated, and pacidusin B exhibited morphological features similar to those of the ferroptosis inducer erastin (Fig. 1G). To further determine the mode of cell death triggered by pacidusin B, several cell death inhibitors were used in combination with pacidusin B (Fig. 1H). The results showed that the decrease in cell viability induced by pacidusin B could be largely reversed by the ferroptosis inhibitor liproxstatin-1 (Lip-1), the iron chelator deferoxamine mesylate (DFOM) and β-mercaptoethanol (β-ME), which could bypass system Xc^−^ by directly converting cystine into cysteine [38]. However, cell viability could also be partially restored by the ROS scavenger N-acetyl-L-cysteine (NAC), the necroptosis inhibitor necrostatin-1 (Nec-1), the apoptosis inhibitor z-VAD-FMK (z-VAD), and the autophagy inhibitor 3-methyladenine (3-MA) [39]. These results suggested that pacidusin B disturbed cellular homeostasis, triggering multiple pathways involved in cell death. However, pathways involved in ferroptosis may play pivotal roles.

### Pacidusin B triggers ferroptotic cell death in HT1080 cells

The finding that the addition of ferroptosis inhibitors largely reversed pacidusin B-induced cell death and the ‘ballooning’ morphological features of cells treated with pacidusin B suggested that pacidusin B triggered ferroptosis. The biochemical hallmarks of ferroptosis, including lipid peroxidation levels, redox status, and LIP levels were evaluated.

**Fig. 2:**
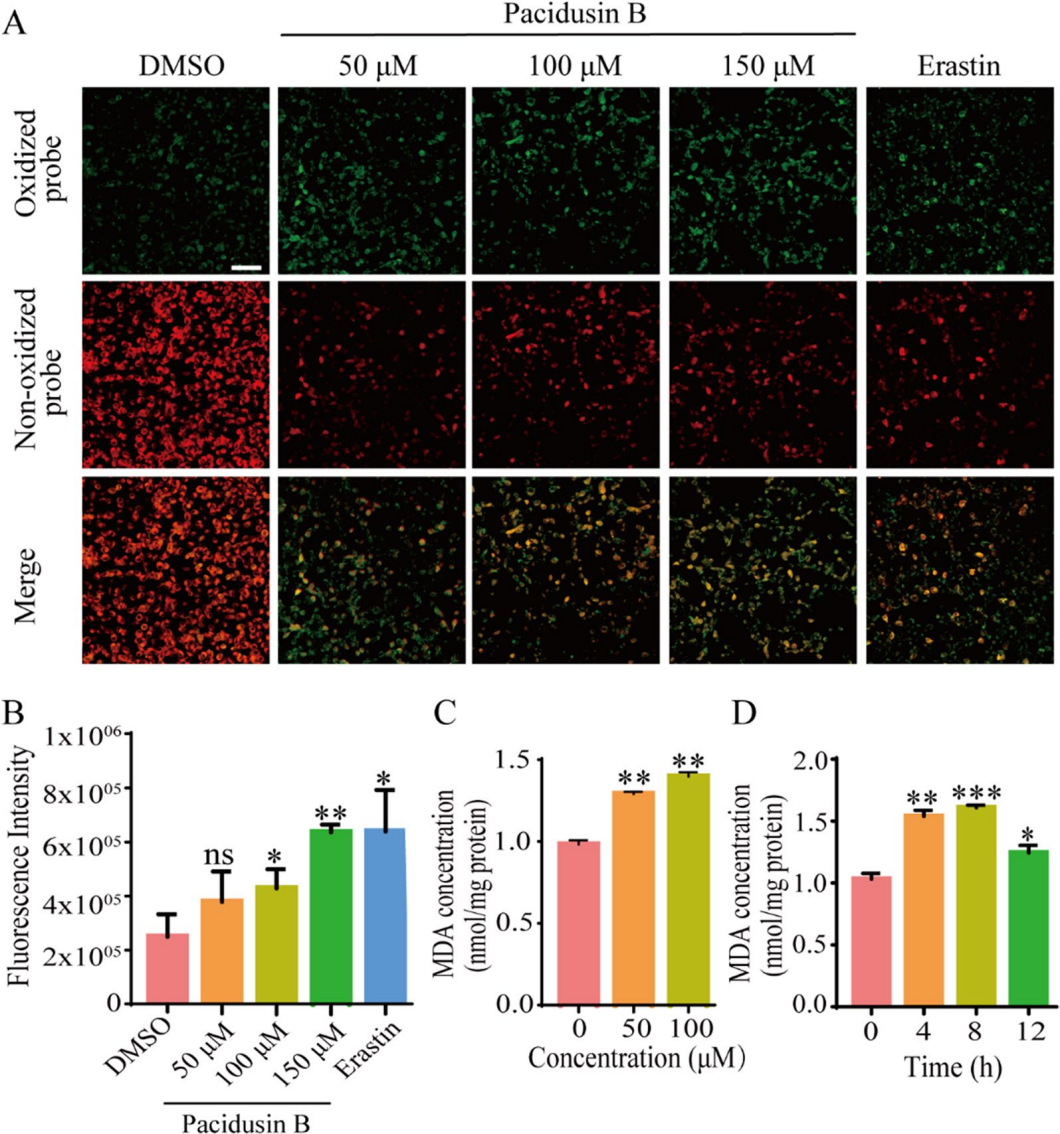
Pacidusin B triggers lipid peroxidation in HT1080 cells. **A** Lipid ROS levels in HT1080 cells treated with DMSO, pacidusin B (50 μM, 100 μM and 150 μM) or erastin (20 μM) for 8 h. Scale bar, 50 μm.** B** The green fluorescence intensity was calculated with ImageJ software to determine the degree of lipid oxidation. **C-D** Changes in the MDA level in HT1080 cells treated with pacidusin B at the indicated concentrations for 10 h or at 100 μM for the indicated times. The data are presented as the mean ± SD. Statistical analysis was performed between the pacidusin B group and the DMSO group. ns, not significant, * *p* < 0.05, ** *p* < 0.01, and ***p < 0.001

Lipid peroxidation is the key biochemical event leading to ferroptosis [40]. The fluorescent probe C11-BODIPY 581/591 is a lipid peroxidation sensor. After treating HT1080 cells with pacidusin B for 8 h, obvious accumulation of intracellular lipid peroxidation was detected in a concentration-dependent manner (Fig. 2A, B). Malondialdehyde (MDA) is one of the most investigated products of PUFA peroxidation [41]. It is the main omega-6 fatty acid lipid peroxidation product. The intracellular MDA level increased in a dose- and time-dependent manner in response to pacidusin B treatment (Fig. 2C, D).

**Fig. 3:**
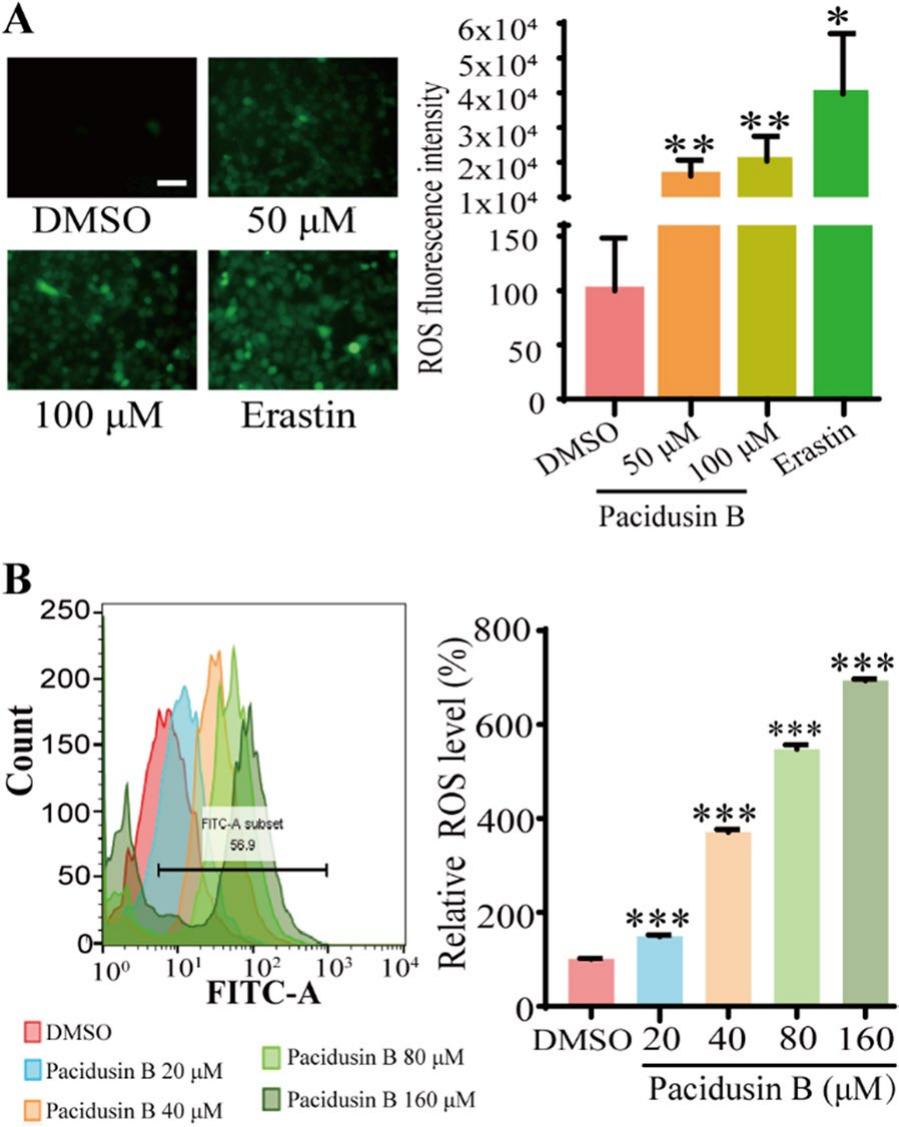
Pacidusin B triggers ROS accumulation in HT1080 cells. **A** Fluorescence microscopy was used to measure the level of ROS in HT1080 cells treated with different concentrations of pacidusin B. Erastin was used as a positive control. Scale bar, 50 μm. **B** The intracellular ROS levels in HT1080 cells treated with different concentrations of pacidusin B were detected via flow cytometry. The data are presented as the mean ± SD. Statistical analysis was performed between the pacidusin B group and the DMSO group. *p < 0.05, **p < 0.01, and ***p < 0.001

ROS also play a key role in lipid peroxidation [42]. Next, whether pacidusin B could trigger ROS accumulation by DCF staining and flow cytometry was investigated. As shown in Fig. 3A, B, ROS increased in a concentration-dependent manner after the addition of pacidusin B. These results are consistent with the finding that the ROS scavenger NAC partially rescued pacidusin B-induced cell death.

**Fig. 4:**
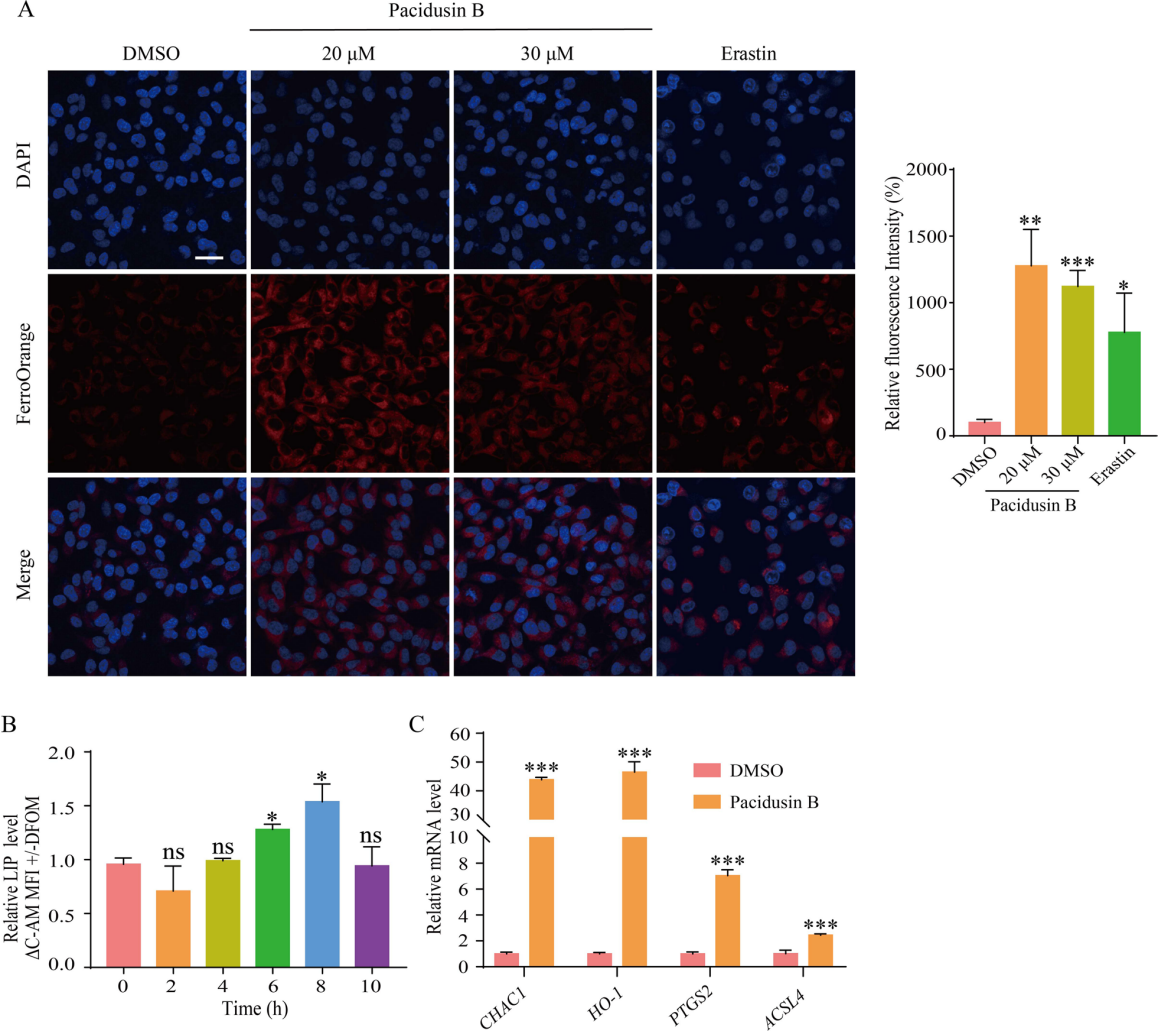
Pacidusin B induces ferroptotic cell death in HT1080 cells. **A** The levels of intracellular Fe^2+^. The cells were stained with FerroOrange and the nuclei were stained with DAPI. **B** The level of LIP in HT1080 cells treated with pacidusin B (60 μM) for the indicated times. **C** The mRNA levels of *CHAC1*, *HO-1*, *PTGS2*, and *ACSL4 *were measured after HT1080 cells were treated with pacidusin B (100 μM) for 4 h. The data are presented as the mean ± SD. ns, not significant, * *p* < 0.05, ** *p* < 0.01, and ***p < 0.001

Another hallmark of ferroptosis is the iron overload [43]. Fe^2+^ can participate in the Fenton reaction to overproduce free radicals, which facilitate the peroxidation of PUFAs. Cells treated with pacidusin B showed increased intracellular Fe^2+^ levels as determined by FerroOrange staining (Fig. 4A). An increased LIP, most of which are composed of Fe^2+^, is another biomarker for ferroptosis [44]. The level of LIP increased within 6 h of pacidusin B treatment (Fig. 4B). In addition, we evaluated genetic hallmarks, including the upregulation of prostaglandin-endoperoxide synthase 2 (*PTGS2*) and chac glutathione specific gamma-glutamylcyclotransferase 1 (*CHAC1*) [45], to validate whether pacidusin B induces ferroptosis. As shown in Fig. 4C, the mRNA levels of heme oxygenase 1 (*HO-1*), *CHAC1*, *PTGS2*, and acyl-CoA synthetase long chain family member 4 (*ACSL4*) increased with pacidusin B treatment. In summary, these results indicate that pacidusin B induces ferroptosis in HT1080 cells.

### Pacidusin B induces ferroptosis by activating the Nrf2-HO-1 signaling pathway.

**Fig. 5:**
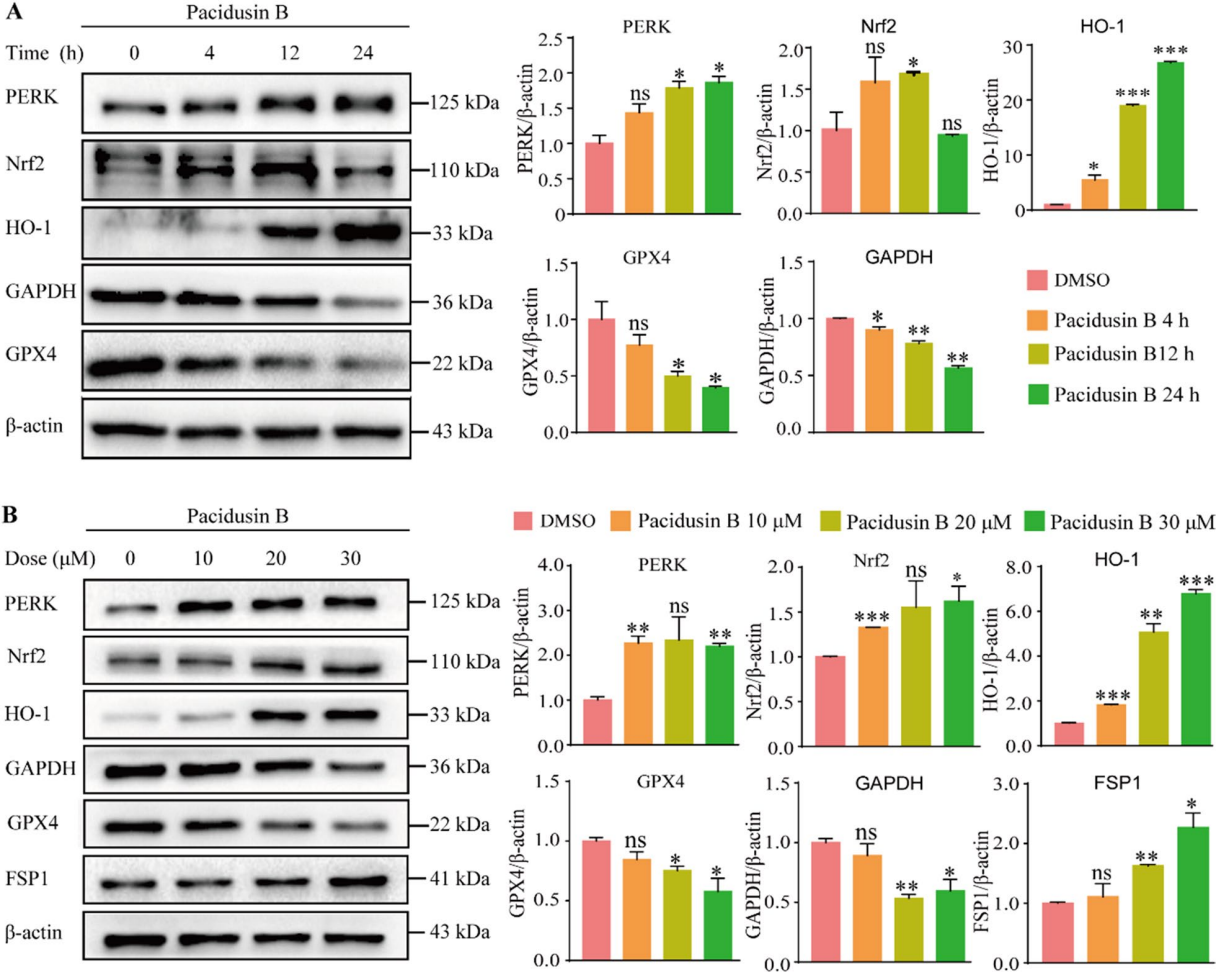
Pacidusin B induces ferroptosis by activating the PERK-Nrf2-HO-1 signaling pathway.** A** Effect of pacidusin B (80 μM) on PERK-Nrf2-HO-1 signaling proteins in HT1080 cells at different time points.** B** Effects of treatment with pacidusin B (10, 20, and 30 μM) for 24 h on ferroptosis-related proteins in HT1080 cells. The data are presented as the mean ± SD. ns, not significant, * *p* < 0.05, ** *p* < 0.01, and ***p < 0.0001

Among the genes upregulated by pacidusin B, the upregulation of HO-1 (up to more than 40-fold compared with that in the DMSO-treated group) was most obvious (Fig. 4C). Previous studies, including those from our group have shown that ferroptosis can be induced through the PERK/Nrf2/HO-1 signaling pathway [25, 46, 47]. Here, another ferroptotic biomarker, CHAC1, which is also associated with ER stress, was also upregulated approximately 40-fold. Therefore, the PERK branch of the ER stress pathway was investigated. PERK-Nrf2 was activated upon pacidusin B treatment (Fig. 5A, B). There are two major ferroptotic death cascades, the GPX4-GSH axis and the FSP1-CoQ10 axis [48]. The levels of GPX4 and FSP1 were checked by western blot analysis. As shown in Fig. 5A&B, the expression of GPX4 decreased, while the expression of FSP1 increased. GAPDH is commonly used as an internal reference gene, but studies have shown that the expression of GAPDH decreases significantly with the progression of ferroptosis [49]. The changes in the expression of GAPDH with pacidusin B treatment is also consistent with previous report. In summary, pacidusin B might induce ferroptosis by activating the Nrf2-HO-1 signaling pathway and by downregulating GPX4.

### Molecular docking of Pacidusin B on xCT

**Fig. 6:**
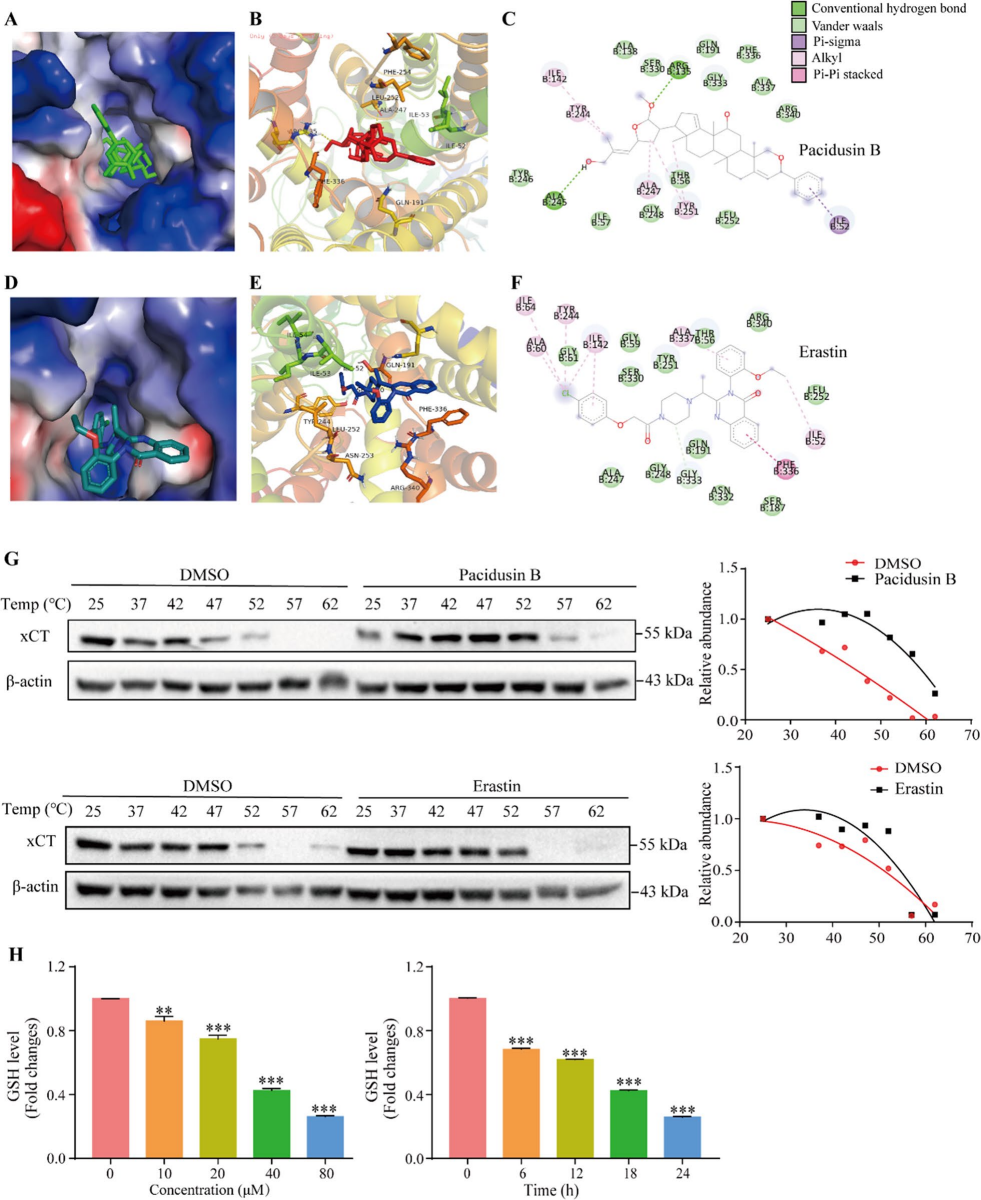
Pacidusin B targets xCT. **A**–**B** Three-dimensional (3D) docking model of the compound pacidusin B, which occupies the binding site of xCT (PDB: 7EPZ). xCT proteins are presented as a cartoon diagram of solid surfaces (**A**). The compound and the amino acid residues involved in the interaction are shown as rods and colored by element, and the hydrogen bonds are shown as yellow dotted lines (B). **C** 2D-docking model of pacidusin B with xCT. **D**–**F** The 3D and 2D docking models of erastin showed that it occupies the binding site of xCT. **G** Representative CETSA blot and CETSA melt curves for xCT. **H** The levels of intracellular GSH. ** *p* < 0.01 and ***p < 0.0001

The well known ferroptosis inducer erastin mainly functions through the inhibition of the cystine-glutamate antiporter system Xc^−^, specifically the light chain xCT. A recent study revealed the binding pocket of erastin on xCT [50], which is located in the intracellular vestibule of xCT. Based on the structure of erastin-bound xCT, molecular docking experiments were carried out to study the interaction between pacidusin B and xCT. The results showed that pacidusin B occupied the same pocket in xCT as erastin, with a binding affinity of -9.5 kcal/mol for pacidusin B compared with a binding affinity of -8.9 kcal/mol for erastin. Three-dimensional (3D) docking models of the pacidusin B-xCT complex and the erastin-xCT complex are shown in Fig. 6A–F. The pacidusin B molecule fits well into the intracellular vestibule of the xCT subunit. By comparing the docking hydrophobic pockets of the xCT subunit between pacidusin B and erastin, many residues in the vicinity of the two are the same, for example, Phe336, Gln191, Ile53, and Leu252. Specifically, pacidusin B formed two conventional hydrogen bonds with the Ala245 and Arg135 residues on xCT, and the benzene ring on pacidusin B formed a pi-sigma interaction with the Ile52 residue. In addition, many van der Waals forces were observed in the predicted complex (Fig. 6B, C). These results indicate that there might be binding interaction between pacidusin B and xCT, which may be the key target of pacidusin B-induced ferroptosis.

Furthermore, whether pacidusin B interacts with xCT was investigated using the cellular thermal shift assays (CETSA) (Fig. 6G). The protein bound to the compound would be more difficult to denature and precipitate due to the improvement in thermal stability. Compared with the DMSO group, pacidusin B and erastin treatments increased the thermal stability of xCT, suggesting that pacidusin B and erastin could bind to xCT. xCT is essential for the uptake of cystine, which is required for the synthesis of the antioxidant GSH. After treating the cells with pacidusin B, the intracellular GSH level decreased in a concentration- and time-dependent manner (Fig. 6H). Taken together, these data indicate that pacidusin B might directly target xCT to induce ferroptosis.

## Discussion

Malignant tumors are among the main factors that seriously threaten human health, and effective anticancer strategies and agents are needed. The identification and characterization of a series of programmed cell death events shed light on novel strategies for cancer therapy. One of the most important types of cell death is ferroptosis. Ferroptosis is a form of iron-dependent cell death that was coined in 2012 [2]. It is a form of cell death different from apoptosis, necrosis and autophagy. Malignant cancer cells are more addicted to iron, which indicates that ferroptosis inducers might be potential therapeutic options for cancer patients [51]. Compounds isolated from plants are important sources of antitumor drugs. Screening for ferroptosis inducers from natural products and illustrating their mechanism of action play indispensable roles in exploring alternative treatments for tumors. In this study, the dichapetalin triterpenoids isolated from the leaves of *P. acidus*, pacidusin A-D, was found to induce ferroptosis in human fibrosarcoma HT1080 cells. First, cells treated with pacidusin B displayed a ‘ballooning’ phenotype. This may be due to lipid peroxidation in ferroptotic cells. When lipid peroxidation occurs, the plasma membrane becomes unstable, accompanied by impaired protein homeostasis and a rearranged cytoskeleton [3]. The morphological characteristics of the pacidusin B-treated cells suggested that the mode of cell death caused by pacidusin B might be ferroptosis. By employing several different cell death inhibitors, pacidusin B-induced cell death can be partially rescued by the necroptosis inhibitor, apoptosis inhibitor, autophagy inhibitor, and ferroptosis inhibitors. However, ferroptosis inhibitors had the most obvious effects on reversing the detrimental effects of pacidusin B, which indicates that iron accumulation and lipid peroxidation play key roles in pacidusin B-induced cell death.

Next, the biochemical and genetic hallmarks of ferroptosis were examined. The biochemical characteristics of ferroptosis include the oxidation of PUFAs, the availability of redox-active iron, and the loss of the capacity to repair lipid peroxide-induced damage. The biochemical characteristics of ferroptosis in pacidusin B-treated cells were observed. In addition, the upregulation of ferroptosis-related genes in response to pacidusin B treatment was detected. *CHAC1* is a ferroptosis-driven gene that plays a key role in regulating glutathione consumption [52]. Notably, *CHAC1* is also an inducible gene involved in ER stress [53]. The mRNA expression level of *CHAC1* was upregulated by pacidusin B treatment, suggesting that ER stress might play a role in pacidusin B-induced ferroptosis. Heme oxygenase 1 (HO-1) is a key regulator of ferroptosis, and it functions as a double-edged sword in ferroptosis [54]. HO-1 catalyzes the catabolism of heme into ferrous iron, carbon monoxide and biliverdin. On the one hand, biliverdin can be converted into bilirubin through biliverdin reductase. Biliverdin and bilirubin act as important antioxidant enzymes in cells by scavenging ROS. On the other hand, ferrous iron is the major pro-oxidant. Excessive ferrous iron disrupts the balance of intracellular redox reactions and eventually leads to ferroptosis [55]. Therefore, when HO-1 is overexpressed, it can exert either pro-ferroptotic or anti-ferroptotic effects. Previously, our group reported that tagitinin C, a sesquiterpene lactone, triggers ferroptosis via HO-1 hyperactivation [25]. In this study, the mRNA levels of *HO-1* were upregulated more than 40-fold while the mRNA expression levels of the other ferroptosis markers* PTGS2* and *ACSL4* were increased 2–8 fold. These results suggested that the hyperactivation of HO-1 induced by pacidusin B might play a pro-ferroptotic role.

Both CHAC1 and HO-1 are ER-related proteins [56]. The ER is a vital organelle in eukaryotic cells and is responsible for the synthesis and folding of secretory and transmembrane proteins [57]. Some exogenous or endogenous factors lead to the accumulation of misfolded or unfolded proteins in the ER, leading to a stress state in the ER called ER stress. To maintain and improve ER function during ER stress, three membrane protein sensors, inositol-requiring 1α (IRE1α), protein kinase R-like ER kinase (PERK), and activating transcription factor 6 (ATF6), are activated [58]. Among them, PERK is phosphorylated in response to ER stress. Activated PERK directly phosphorylates Nrf2. Nrf2 and Keap1 are dissociated and released, thereby activating the Nrf2-HO-1 signaling pathway [59, 60]. Here, we showed that pacidusin B treatment led to the upregulation of proteins in the PERK-Nrf2-HO-1 axis, which indicated that pacidusin B might trigger ER stress-mediated ferroptosis.

One of the molecular features of ferroptosis is the loss of lipid peroxide repair capacity via the system Xc^−^/GSH/GPX4 axis or ferroptosis suppressor protein 1 (FSP1)/CoQ10/NAD(P)H axis. GPX4 mainly catalyzes the reduction of lipid hydroperoxides, protecting cells from oxidative stress. FSP1 catalyzes the NAD(P)H-dependent reduction of CoQ10 to ubiqunol-10, which is a lipophilic radical-trapping antioxidant that prevents lipid oxidative stress [61]. FSP1 acts in parallel to GPX4 to suppress ferroptosis. Here, pacidusin B treatment of HT1080 cells significantly reduced the protein expression level of GPX4 while increasing the protein expression of FSP1. These results suggested that pacidusin B might inhibit GPX4 activity by downregulating GPX4 protein levels, and that FSP1 might be upregulated to alleviate lipid oxidative stress.

Moreover, the results of computational molecular docking showed that pacidusin B fit well into the intracellular vestibule of xCT, which is the same pocket in xCT as erastin, suggesting that the target of pacidusin B-induced ferroptosis may be similar to that of erastin. The results from the CETSA indicated that pacidusin B might directly bind to xCT as erastin does. The decreased intracellular GSH levels also support this hypothesis. Pacidusin B might induce ferroptosis via xCT inhibition.

## Conclusions

In summary, pacidusin B, a dichapetalin triterpenoid from *P. acidus*, triggered ferroptosis in HT1080 cells. Mechanistically, pacidusin B might induce ferroptotic cell death via oxidative stress-mediated ER stress, hyperactivation of the HO-1 signaling pathway and inhibition of GPX4. Moreover, pacidusin B occupied the same pocket in xCT as erastin did, suggesting that pacidusin B might directly inhibit xCT. The characterization of pacidusin B-induced ferroptosis broadens the understanding of dichapetalin triterpenoids as ferroptosis inducers. Elucidation of the mechanisms underlying ferroptosis induced by pacidusin B is of great significance for exploiting dichapetalin triterpenoids as novel ferroptosis inducers for cancer therapy.

## Materials and methods

### Cell culture

The fibrosarcoma HT1080 cell line was purchased from the China Center for Type Culture Collection (CCTCC). The cells were cultured in DMEM supplemented with 10% fetal bovine serum and 1% penicillin/streptomycin at 37 °C in a humidified atmosphere containing 5% CO_2_. The cells were used during their logarithmic growth phase.

### Cell viability assay

The 3-(4,5-dimethylthiazol-2-yl)-2,5-diphenyltetrazolium bromide (MTT) assay was used to determine cell viability. The cells were cultured in 96-well plates at a density of 5 × 10^3^ cells per well and incubated in 100 µL of culture medium at 37 °C. Then, various concentrations of pacidusin A-D (purity > 95%) were added and incubated for 24 h. Afterwards, 20 μL of MTT solution (5 mg/mL in PBS, Sigma) was added to each well, and the cells were further incubated for 4 h. After removing the MTT medium, 100 μL of DMSO was added to dissolve the precipitate. The absorbance at 490 or 450 nm was measured with a Biotek ELx808 absorbance plate reader.

### Detection of lipid peroxidation levels

The level of cellular lipid peroxidation was detected by a C11-BODIPY 581/591 (Cayman Chemical, Michigan). After the cells were treated with the test compounds, 5 μM of C11-BODIPY 581/591 dye was added to fresh medium and incubated in the dark at 37 °C for 30 min. The C11-BODIPY 581/591 dye solution was removed, and then the cells were washed twice with PBS to remove the excess probe. Lipid ROS fluorescence was captured by a Leica laser scanning confocal microscope. Three random fields per sample were quantified using ImageJ software.

### Measurement of malondialdehyde (MDA) levels

After the HT1080 cells were treated with pacidusin B, the relative MDA concentration in the cell lysate was evaluated using an MDA assay kit (Beyotime, Beijing) according to the manufacturer’s instructions. The content was determined at 532 nm by a microplate reader.

### Measurement of ROS levels

Intracellular ROS levels were quantified by measuring the fluorescence of DCF using a Reactive Oxygen Species Assay Kit (MeilunBio, Dalian). Briefly, after treatment with pacidusin B, the HT1080 cells were incubated with 20 μM DCFH-DA at 37 °C in the dark for 30 min. The cells were rinsed with 3 mL of PBS per well to remove residual DCFH-DA dye. After that, 0.5 mL of PBS containing 5% FBS was added to each well to prevent the cells from drying, and images were captured with a BioTek Cytation imaging reader. In addition, ROS levels were detected via flow cytometry on a BD FACS Celesta flow cytometer.

### Assay for glutathione levels

The levels of GSH in HT1080 cells were measured using monochlorobimane (MBCL, Sigma) according to the manufacturer’s instructions. The treated cells were incubated with MBCL (32 μM) in PBS at 37 °C in the dark for 30 min, after which the OD was detected using a microplate reader to calculate the GSH concentration at different concentrations and times.

### ***Measurement of the intracellular Fe***^***2***+^***levels***

For detecting the LIP levels, HT1080 cells were plated into 96-well plates and grown overnight. After the cells were treated with the compound, they were washed once with PBS. At 37 °C, the cells were incubated with 0.2 μM calcein-acetoxymethyI ester (calcein-AM, GLPBio) for 30 min, and then washed once with 100 μL of PBS per well. Then, 100 μL of deferiprone was added to each well and incubated at 37 °C for 30 min or without treatment, after which the OD was detected by a microplate reader.

Free Fe^2+^ levels in the cytoplasm was detected using FerroOrange (Dojindo, F374). HT1080 cells were treated with pacidusin B or erastin for 8 h, and then incubated with 1 μM FerroOrange and DAPI for 30 min at 37 °C. The images were captured using Leica laser scanning confocal microscope directly at the end of staining. The fluorescence intensity was quantified using Image J software.

### Quantitative real-time polymerase chain reaction (qPCR) analysis

After the HT1080 cells were treated with pacidusin B (100 μM) for 4 h, total RNA was extracted with TRIzol reagent, and the RNA concentration was determined. 1 μg of RNA was used for reverse transcription to synthesize cDNA. After reverse transcription, qPCR was performed according to the manufacturer's instructions. The sequences of the primers used for qPCR were as follows: *CHAC1*: 5′-GAACCCTGGTTACCTGGGC-3′ and 5′-CGCAGCAAGTATTCAAGGTTGT-3′; *HO-1*: 5′‐AAGACTGCGTTCCTGCTCAAC‐3′ and 5′‐AAAGCCCTACAGCAACTGTCG‐3′; *PTGS2*: 5′-CTGGCGCTCAGCCATACAG‐3′ and 5′‐CGCACTTATACTGGTCAAATCCC-3′; *ACSL4*: 5′-CATCCCTGGAGCAGATACTCT-3′ and 5′-TCACTTAGGATTTCCCTGGTCC-3′; *actin*: 5′-GATCTGGCACCACACCTTCT-3′ and 5′-GGGGTGTTGAAGGTCTCAAA-3′.

### Western blot assay

The treated cells were washed once with cold PBS, and lysed with RIPA lysis buffer on ice for 30 min. The lysates were vertebrated every 5 min to accelerate lysis. After centrifugation at 12000 rpm, and 4 °C for 15 min, the supernatant was collected, and the protein concentration was determined using a BCA assay kit. The proteins were electrophoresed on a 12% SDS‒PAGE gel, transferred to a PVDF membrane, blocked in 5% milk for 1 h and incubated with primary antibody at 4 °C overnight. The primary antibodies used were as follows: anti-PERK (Cell Signaling Technology, Cat# 5683), anti-Nrf2 (Abcam, Cat# ab137550), anti-HO-1 (Abcam, Cat# ab52947), anti-GPX4 (Abcam, Cat# ab125066), anti-GAPDH (Proteintech, Cat# 60004-1-Ig), anti-FSP1 (Proteintech, Cat# 20886-1-AP), anti-xCT (MCE, Cat#YA006) and anti-actin (Proteintech, Cat# 66009-1-Ig). The membrane was washed three times with PBST for 10 min each time. Next, the membrane was incubated with the secondary antibody at room temperature for 2 h. Finally, the membrane was washed three times with PBST for 10 min each time, incubated with an appropriate amount of ECL chemiluminescence solution, and developed with a Bio-Rad imaging system.

### Molecular docking

AutoDock Vina software was used to simulate xCT and compound molecular docking. The published 3D crystal structure of xCT (PDB: 7EPZ) was downloaded from the RCSB protein database (https://www.rcsb.org/) [62, 63], and the three-dimensional (3D) structure of pacidusin B was drawn with ChemDraw software. In the docking calculation, the ligands and crystal water molecules were removed, and the best matching posture was selected according to the docking score. PyMOL software was used for further visual graphics preparation.

### Cellular thermal shift assays

Cells were treated with pacidusin B or erastin for 24 h, and then the cells were collected and heated individually at different temperatures (25–62 °C) for 3 min. Cell lysates were obtained by repeated freeze-thawing in liquid nitrogen. The cell lysates were centrifuged and the supernatants were collected. Western blot analysis was carried out to detect the levels of candidate proteins.

### Statistical analysis

All the experiments were conducted at least three times, and the data are presented as the mean ± SD. All the data were collected and analyzed using ImageJ and Prism 8.02 software (GraphPad). *p* values were analyzed by using Student’s t-test. *p* < 0.05 was considered statistically significant.

## Data Availability

All the data are available in the main text. All the data generated in this study can be obtained from the corresponding authors upon reasonable request.
